# Anoikis-related biomarkers PARP1 and SDCBP as diagnostic and therapeutic targets for asthma

**DOI:** 10.1038/s41598-025-09979-9

**Published:** 2025-07-09

**Authors:** Li-jie Yang, Na-na Song, Ni-shan Deng, Miao-juan Zhu, Qing-qing Li, Si-si Huang, Xiu Shi, Zhen-Hong Hu, Han-Xiang Nie

**Affiliations:** 1 Department of Respiratory and Critical Care Medicine, Renmin Hospital of Wuhan University, Wuhan, 430060 China; 2 Department of Clinical Laboratory, Renmin Hospital of Wuhan University, Wuhan, 430060 China; 3 Department of Respiratory and Critical Care Medicine, General Hospital of Center Theater of PLA, Wuhan, 430070 China

**Keywords:** Asthma, Anoikis, Biomarkers, Machine learning, Genetics, Immunology, Respiratory tract diseases

## Abstract

This study aims to explore the association between anoikis-related genes (ARGs) and asthma. The dataset GSE143303 for asthma were sourced from the GEO database, while ARGs were retrieved from the Harmonizome web portal and the GeneCards database. Differentially expressed genes (DEGs) identification and GO, KEGG enrichment analysis were performed to reveal potential biological pathways. To identify hub anoikis-related DEGs (hub ARDEGs), we employed WGCNA and machine learning methods including LASSO and Random Forest. Additionally, we constructed risk prediction nomogram model and ROC curves to evaluate the asthma diagnostic value of hub ARDEGs. SsGSEA immune infiltration analysis was used to analyze the role of hub ARDEGs in the asthma immune microenvironment. Finally, miRNAs and transcription factors (TFs) interacting with these hub ARDEGs were investigated. DEGs of ARGs between asthma and healthy controls, along with WGCNA, led to the identification of six ARDEGs. GO and KEGG analyses revealed that these ARDEGs were primarily involved in the apoptotic signaling pathway and adherens junctions. Machine learning methods further narrowed down the six ARDEGs to two hub ARDEGs: PARP1 and SDCBP, which were significantly upregulated in asthma and validated using the GSE147878 and experimental models. Based on these two hub ARDEGs, a risk prediction model for asthma was developed, demonstrating strong diagnostic potential and tissue specificity in endobronchial biopsies. Immune analysis revealed variations in immune cell infiltration within asthma samples correlated with hub ARDEGs. Additionally, the miRNA-TF-mRNA interaction network of hub ARDEGs highlights the complexity of the regulatory process. The process of anoikis, immune dysregulation, and asthma are closely interconnected. The anoikis-related biomarkers PARP1 and SDCBP may serve as diagnostic markers and therapeutic targets for asthma.

**Supplementary Information:**

The online version contains supplementary material available at 10.1038/s41598-025-09979-9.

## Introduction

Asthma is a prevalent and multifactorial respiratory disease that affects approximately 300 million people globally, causing about 1,000 deaths per day^1^. Despite the availability of traditional treatments like inhaled corticosteroids and bronchodilators, many patients remain inadequately controlled^2^. One reason for this is that these therapies mainly manage symptoms without addressing the underlying genetic and molecular mechanisms. Although biologics targeting Type 2 inflammation, such as anti-IgE, anti-IL-5, and anti-IL-4/IL-13 antibodies, have improved severe asthma management, they still do not fully address the complex genetic and molecular mechanisms involved^3,4^. Therefore, investigating hub genes and their mechanisms in asthma is crucial for timely identifying and managing disease progression.

Anoikis is a specialized form of programmed cell death that occurs when cells are detached from the extracellular matrix (ECM). This process, triggered by the disruption of integrin junctions, prevents abnormal cells from proliferating or adhering to inappropriate matrices^5^. Anoikis plays a significant role in the pathogenesis of asthma by modulating airway inflammation and driving remodeling processes. Matrix metalloproteinase 9 (MMP9) disrupts cell-cell and cell-matrix adhesion, activating cell death pathways. This promotes anoikis in airway epithelial cells, contributing to airway remodeling in asthma^6^. In severe asthma, excessive epithelial proliferation can disrupt contact with the basement membrane, triggering anoikis in airway epithelial cells. This dysregulated apoptosis, alongside increased proliferation, results in a thicker, remodeled epithelium^7^. Thus, anoikis is crucial in the pathogenesis of asthma, although the specific molecules and mechanisms involved remain unclear, warranting further investigation into the relationship between asthma and anoikis.

In this study, we aim to explore anoikis-related characteristic genes in asthma, propose a new diagnostic model for asthma, and analyze the immune infiltration patterns and the miRNA-TF-mRNA interaction network of these characteristic genes in asthma.

**Fig. 1:**
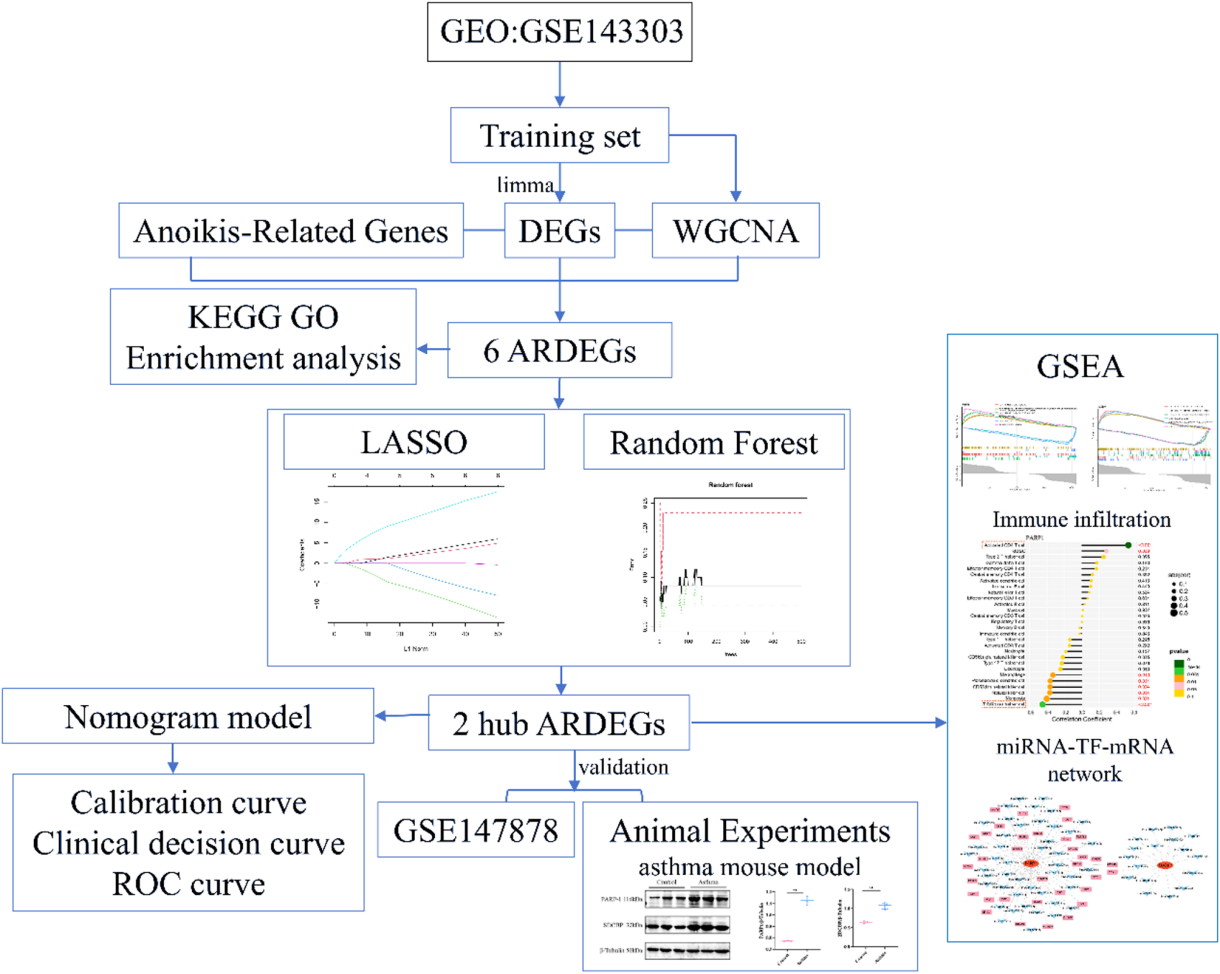
The flowchart of this study.

## Materials and methods

### Data acquisition

Asthma-related transcriptome datasets were obtained from the Gene Expression Omnibus (GEO) database (https://www.ncbi.nlm.nih.gov/geo/). The Selection criteria were as follows: Homo sapiens samples; expression profiling by array; asthma-related tissues; studies including both asthma and control groups (*n* ≥ 5 per group); and data availability on respiratory tissues. The GSE143303 dataset^8^, containing 13 control endobronchial biopsies tissues and 47 asthma endobronchial biopsies tissues, served as the training cohort. The GSE147878, GSE64913, GSE137268 and GSE27876 datasets served as validation cohorts. To ensure consistency across platforms, the probe IDs in the matrix expression profiles were converted to standardized gene symbols using the mapping provided in the corresponding GPL files. Data from different platforms were then normalized using R’s normalizeBetweenArrays function to make the datasets comparable across samples.

A total of 509 anoikis-related genes (ARGs) were retrieved from the GeneCards database (https://www.genecards.org/) and the Harmonizome web portal (https://maayanlab.cloud/Harmonizome/) (Table S1). The detailed information about these datasets is provided in Table 1. The analysis workflow for this study is illustrated in Fig. 1.

**Table 1:** Detailed information on datasets used in the study.

DataSets	Platforms	Sample size		Organism	Tissue
		Control asthma			
GSE143303	GPL10558	13	47	Homo sapiens	Endobronchial biopsies
GSE147878	GPL10558	13	59	Homo sapiens	Endobronchial biopsies
GSE64913	GPL570	42	28	Homo sapiens	Epithelial brushings
GSE137268	GPL6104	15	54	Homo sapiens	Induced sputum
GSE27876	GPL6480	5	10	Homo sapiens	Peripheral blood
Anoikis related genes	Harmonizome + GeneCards	509	–	Homo sapiens	–

### Identification and enrichment analysis of differentially expressed genes

The original data were processed in R software. The “limma” program was used to evaluate the differentially expressed genes (DEGs) between the asthma and control groups, with filtering criteria of |log2FC| > 0.40 and P.Val < 0.05.A volcano plot was used to depict DEGs, and the “pheatmap” and “ggplot2” packages were used to create a clustered heatmap. The “clusterProfiler” package was used to perform pathway enrichment analyses for the Kyoto Encyclopedia of Genes and Genomes (KEGG)^9,10^ and Gene Ontology (GO).

### Weighted gene co-expression network analysis (WGCNA)

WGCNA^11^ was performed using the R package to build a gene co-expression network and identify asthma-related modules. Initially, genes with a lower median absolute deviation (MAD) were removed. The soft thresholding power (β) was selected based on a fit index > 0.90. The adjacency matrix was converted into a topological overlap matrix (TOM). Gene modules were determined through unsupervised clustering and hierarchical clustering. Then, modules were merged based on highly correlated eigengenes. Finally, six gene modules were identified, and their correlation with asthma was visualized using a heatmap. Significant modules were further analyzed by assessing module membership (MM) and gene significance (GS) scores.

### Identification of ARDEGs and correlation analysis of ARDEGs

To identify anoikis-related differentially expressed genes (ARDEGs), we intersected the DEGs with the ARGs and WGCNA genes. A Venn diagram was used to visualize the overlap of these gene sets. The correlation between ARDEGs was analyzed using the Pearson correlation coefficient. Strong correlations (|cor| > 0.7) were considered for further investigation. A heatmap was used to display the pairwise correlations, helping to identify potential regulatory interactions among the ARDEGs.

### Identification of hub ARDEGs

Hub ARDEGs were identified using the least absolute shrinkage and selection operator (LASSO) regression and Random Forest (RF) methods. LASSO regression is a widely used data mining method^12^. ARDEGs were incorporated into the diagnostic model using “glmnet” package, setting the glmnet function value to 1. The best λ value was determined through 5 cross validations, leading to the identification of anoikis signature genes. Recursive Feature Elimination (RFE) in the RF algorithm, a supervised machine learning technique^13^, identified anoikis signature genes with a relative importance greater than 1 and the decision tree set to 500. The intersection of the two machine learnings with ARDEGs was defined as hub ARDEGs using the “Venn” package. The diagnosis accuracy of the hub ARDEGs for asthma was evaluated using receiver operating characteristic (ROC) analysis on both the LASSO and RF models.

### Construction of nomogram

In order to assess the diagnostic performance of the distinctive genes, the “rms” package was utilized to create a nomogram. To evaluate the nomogram’s prediction accuracy, a calibration curve was created. Each gene in the nomogram was assigned a corresponding score, and the total score was used to predict the risk of asthma. The “rmda” program was used to perform decision curve analysis in order to evaluate the net benefit of the nomogram forecasts.

### Evaluation of signature genes and nomogram

Two distinctive genes’ expression levels were compared across the training cohort (GSE143303) and validation cohorts (GSE147878, GSE64913, GSE137268, and GSE27876). The diagnostic performance of the genes and nomogram was further evaluated through ROC curves generated using the “pROC” package.

### Construction of the mouse asthma model

We purchased female 6-week BALB/c mice from Wuhan University’s Animal Experimental Center in China. To establish the asthma mouse model, mice were sensitized by intraperitoneal injection of 20 µg OVA (Sigma) dissolved in 2 mg Imject Alum (Thermo Scientific) in 200 µL PBS on days 0 and 14. From days 21 to 23, the mice were anesthetized with an intraperitoneal injection of 1% pentobarbital sodium solution (60–80 mg/kg), followed by challenge with 100 µg OVA in 50 µL PBS via intranasal administration. Mice were euthanized by CO_2_ asphyxiation, with death defined 30 min after cessation of breathing^14,15^.

The animal study followed the Guide for the Care and Use of Laboratory Animals, in accordance with ARRIVE guidelines (https://arriveguidelines.org), and was approved by the Wuhan University Ethics Committee (Approval No. WP20230530). All experimental procedures were performed in strict accordance with the relevant guidelines and regulations, and all methods were carried out in compliance with institutional and national ethical standards.

### RT-PCR

Lung tissue RNA was extracted using TRIzol reagent (G3013, Servicebio). FastKing gDNA Dispelling RT SuperMix (KR118, TIANGEN Biotech, China) was used to generate cDNA, and SYBR Green mix (CW0956M, CWBIO, China) was used for RT-PCR.RNA expression levels of hub AREDGs were quantified. Table 2 lists the primer sequences.

**Table 2:** The primer sequences of RT-PCR primers.

Primer (mouse)	Sequence (5’ to 3’)
PARP1-F	AGTATGCCAAGTCCAACAGAAAGTAGG
PARP1-R	CCAGCGGGTCAATCATGCCTAGC
SDCBP-F	GCTTGAAGGATGCTCAGATTGC
SDCBP-R	AGGAATGGTGTGATCCATCAGG

### Western blot

Lung tissue proteins were extracted and separated using 10% SDS-PAGE, followed by transfer onto a PVDF membrane with a thickness of 0.45 μm. The membrane was blocked for one hour at room temperature using 5% skim milk, and then it was treated for the entire night at 4 °C with primary antibodies that targeted β-Tubulin (60004-1-Ig, Servicebio, China), PARP1 (12118-1-AP, Proteintech, China), and SDCBP (22399-1-AP, Proteintech, China). Secondary antibodies were applied the following day and left at room temperature for an hour. An enhanced ECL detection kit (HYC0316, HY CEZMBIO, China) was then used to find protein bands, and ImageJ software was used to quantify them.

### Immune cell infiltration analysis

Single sample gene set enrichment analysis (ssGSEA), an extension of the GSEA method^16^, is used to evaluate immune infiltration across 28 immune cell types. Enrichment scores for normal and asthmatic samples were calculated with the “GSVA” package, with results visualized using the “vioplot” package. Spearman correlation analysis was conducted to assess relationships between hub ARDEGs and immune cells.

### Gene set enrichment analysis (GSEA)

Single-gene GSEA was conducted on hub ARDEGs to identify significant GO and KEGG pathways distinguishing asthma and control groups^17^. Pathway enrichment was carried out by the “clusterProfiler” package with the gseGO and gseKEGG functions. When the p-value was less than 0.05, the gene set was deemed substantially enriched.

### The miRNA-TF-mRNA regulatory network construction

MiRTarBase was used to predict miRNAs targeting hub ARDEGs^18^, while the Enrichr database identified associated transcription factors (TFs) with a p-value < 0.05. Next, Cytoscape (version 3.9.0) was used to build and show miRNA-TF-mRNA regulatory networks.

### Statistical analysis

For all statistical studies, R software (version 4.2.3) was used. The Student’s t-test or the Wilcoxon test were used to evaluate differences between two groups. Relationships between variables were examined using the Pearson or Spearman correlation tests. A significance level of *P* < 0.05 was deemed statistically significant.

## Results

### Identification and enrichment analysis of DEGs in asthma

**Fig. 2:**
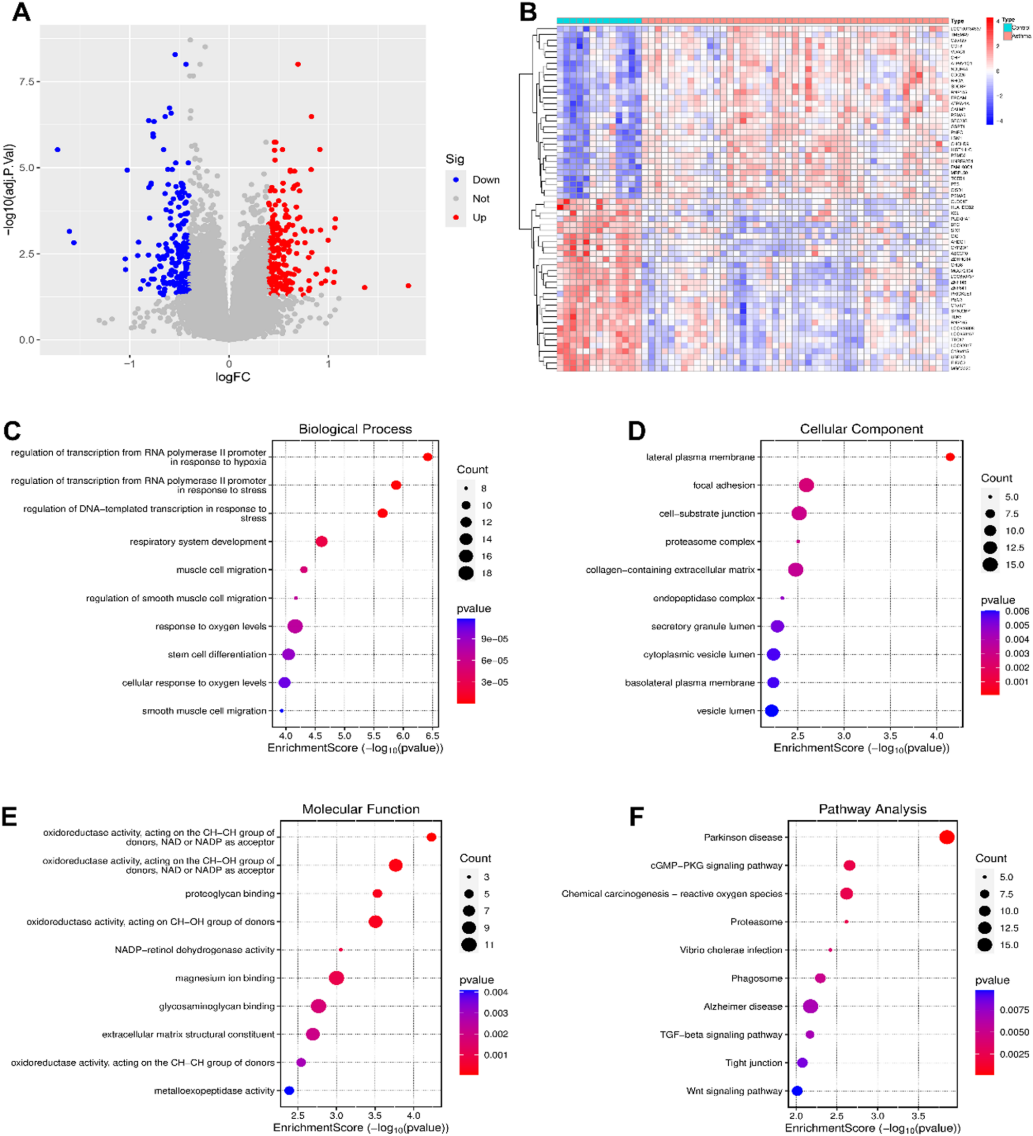
Screening of DEGs and asthma-related DEGs. (**A**) Volcano plot illustrating upregulated (red) and downregulated (blue) DEGs. (**B**) Heatmap displaying hierarchical clustering of DEGs between asthma and control groups. (**C–E**) Bubble plots representing GO enrichment analysis of DEGs, including Biological Processes (BP), Cellular Components (CC), and Molecular Functions (MF). (**F**) Bubble plot depicting KEGG enrichment analysis of DEGs. Figure (**F**) is cited from the source: www.kegg.jp/kegg/kegg1.html.

Limma analysis of the training dataset GSE143303 revealed a distinct gene expression profile between the control and asthma groups, identifying 417 DEGs (216 upregulated and 201 downregulated) (Fig. 2A, Table S2). Hierarchical clustering was visualized through a heatmap (Fig. 2B). GO analysis showed significant enrichment in Biological Processes (BP) like “regulation of transcription from RNA polymerase II promoter in response to hypoxia” and “regulation of DNA-templated transcription in response to stress” (Fig. 2C); Cellular Components (CC) like “lateral plasma membrane,” “cell-substrate junction,” and “focal adhesion” (Fig. 2D); and Molecular Functions (MF) like “oxidoreductase activity” and “proteoglycan binding” (Fig. 2E). KEGG enrichment analysis revealed significant pathways, including the “cGMP-PKG signaling pathway,” “Chemical carcinogenesis - reactive oxygen species,” and “Proteasome” (Fig. 2F) (Table S3–S6).

### Identification of gene modules associated with asthma

**Fig. 3:**
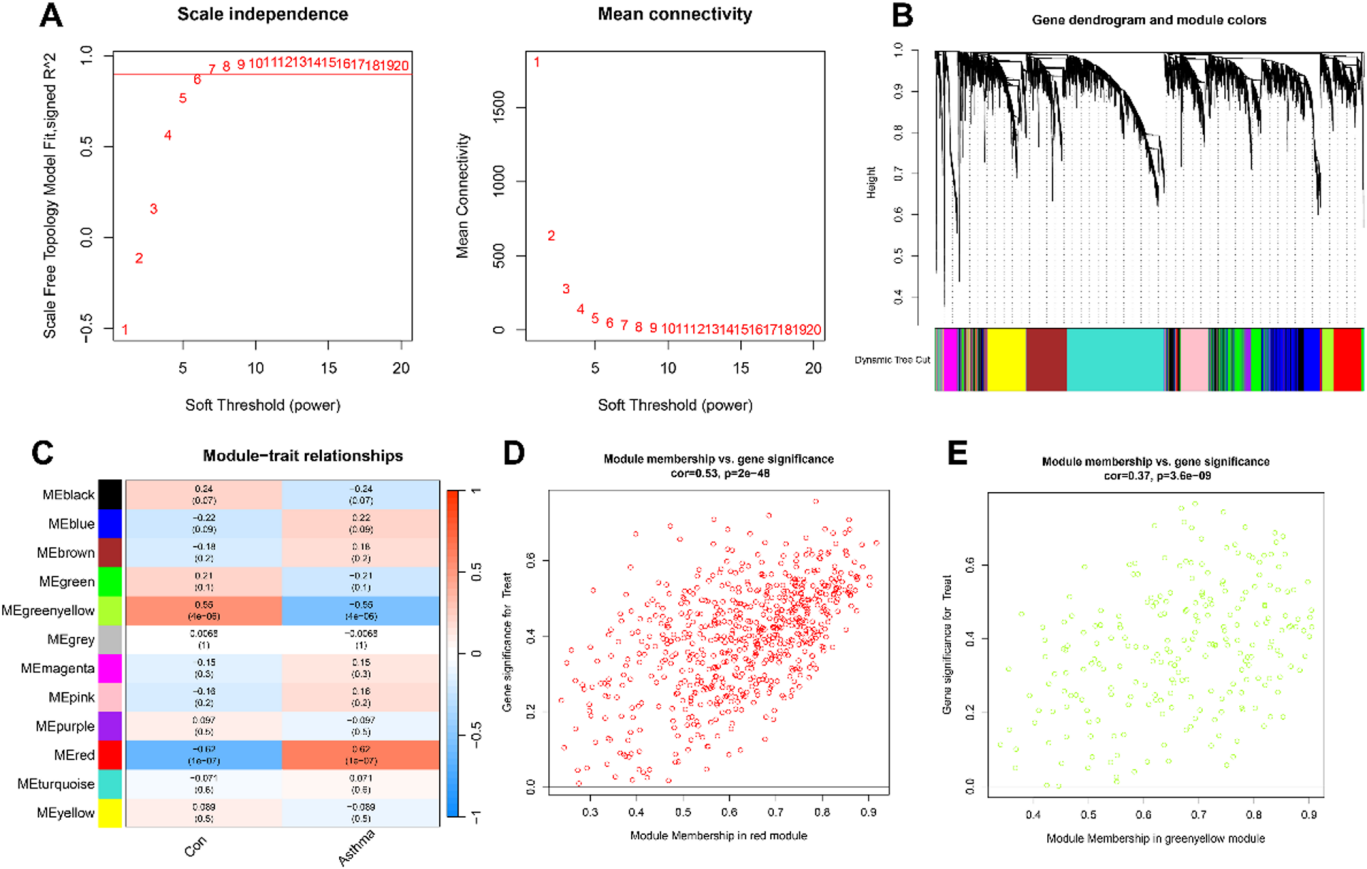
Identification of gene modules associated with dilated asthma using WGCNA. (**A**) Selection of optimal soft thresholding power (β) based on a scale-free fit index (left) and mean connectivity (right) for varying powers. (**B**) Gene dendrogram illustrating modules associated with asthma, represented by distinct colors. (**C**) Correlation heatmap demonstrating relationships between gene modules and asthma status. (**D,E**) Scatter plots showing correlations between module membership (MM) and gene significance (GS) in the red and greenyellow modules.

**Fig. 4:**
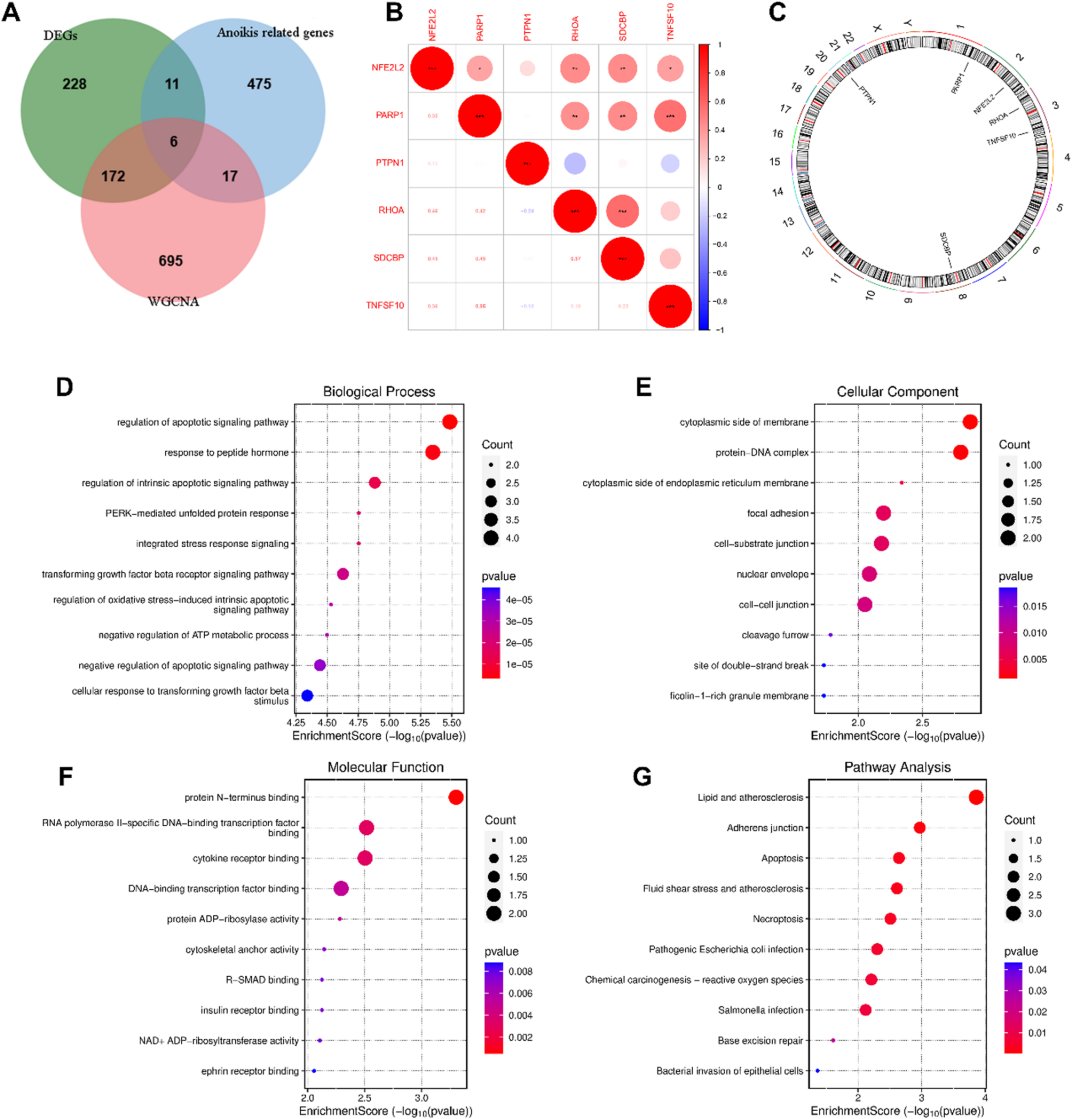
Correlation and enrichment analysis of ARDEGs. (**A**) Venn diagram depicting the intersection of DEGs, WGCNA genes, and Anoikis-related genes. (**B**) Correlation analysis of ARDEGs, *P < 0.05, **P < 0.01, ***P < 0.001. (**C**) Chromosomal distribution of six ARDEGs. (**D–F**) Bubble plots of GO enrichment analysis of ARDEGs, encompassing BP, CC, and MF. (**G**) Dot plot illustrating the top 10 KEGG entries for ARDEG enrichment. Figure (**G**) is cited from the source: www.kegg.jp/kegg/kegg1.html.

Using WGCNA, gene modules significantly associated with asthma were identified. The optimal soft thresholding (R² = 0.9) established a scale-free network (Fig. 3A). After merging highly correlated eigengenes, six modules were identified (Fig. 3B), and module-trait relationships were assessed (Fig. 3C). The red module (651 genes, *r* = 0.62, *P* = 1e-07) showed the strongest positive correlation with asthma, while the greenyellow module (239 genes, *r* = -0.55, *P* = 4e-06) showed the strongest negative correlation. Scatter plots confirmed biological relevance, with significant correlations between GS and MM for both modules (red: cor = 0.58, *P* = 2e-48; greenyellow: cor = 0.37, *P* = 3.6e-09) (Fig. 3D-E, Table S7). The intersection of 890 genes from these modules with DEGs and ARGs identified six ARDEGs (Fig. 4A, Table S8).

### Correlation and enrichment analysis of ARDEGs

Six ARDEGs were identified by intersecting DEGs, WGCNA genes, and ARGs (Fig. 4A). Pearson correlation analysis revealed relationships among these genes (Fig. 4B), and their chromosomal locations were mapped (Fig. 4C, Table S9). GO enrichment analysis showed that ARDEGs were enriched in BP like “regulation of apoptotic signaling pathway,” “response to peptide hormone,” and “regulation of intrinsic apoptotic signaling pathway,” CC like “cytoplasmic side of membrane,” and MF like “protein N-terminus binding” (Fig. 4D-F). KEGG pathway analysis highlighted key pathways involving ARDEGs, such as “Lipid and atherosclerosis,” “Adherens junction,” “Apoptosis,” “Fluid shear stress and atherosclerosis,” and “Necroptosis” (Fig. 4G) (Table S10-S13).

### Identification and validation of hub ARDEGs

**Fig. 5:**
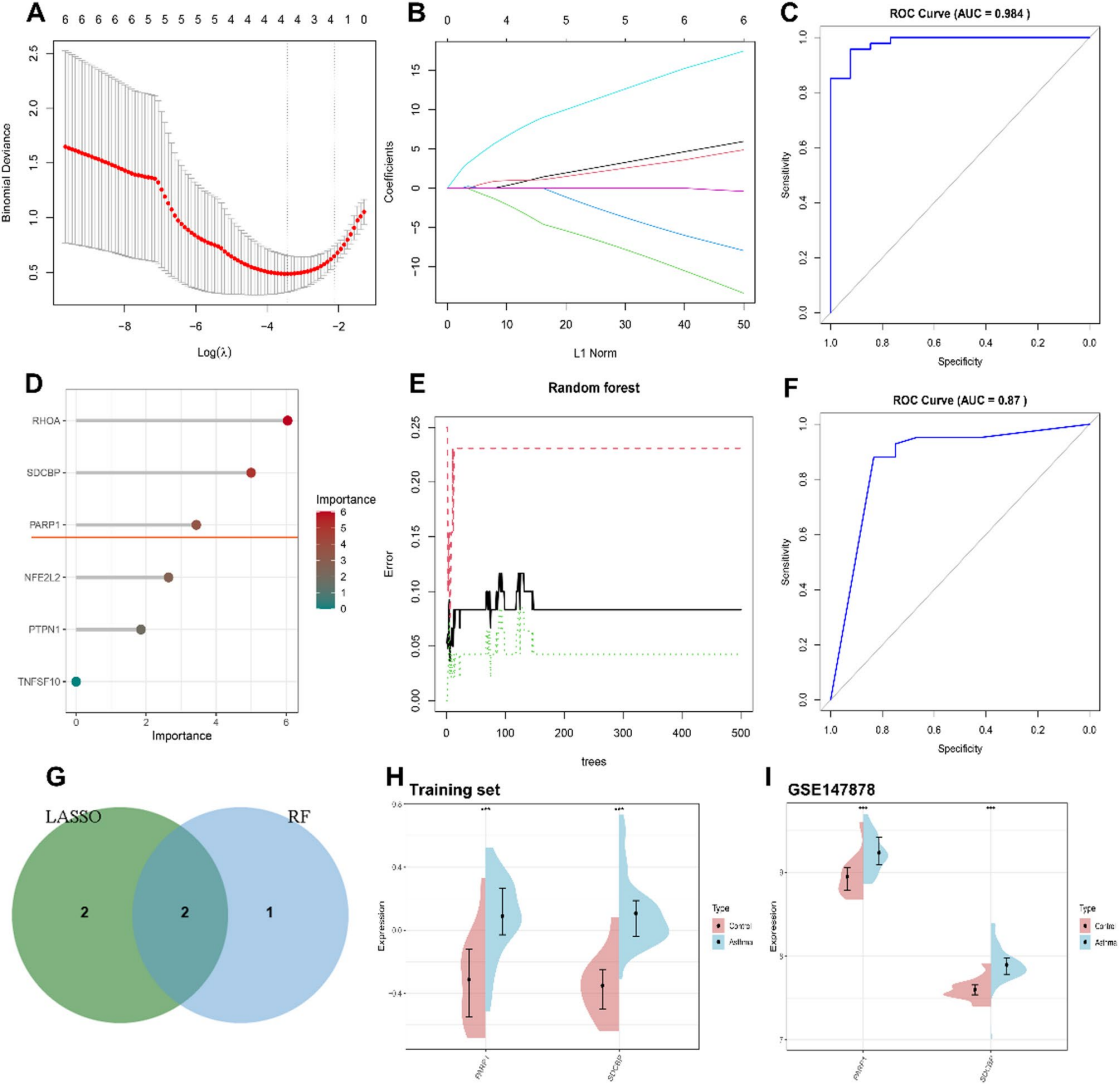
Identification of hub ARDEGs by machine learning and Evaluation of gene expression. (**A**) LASSO coefficient analysis, with vertical dashed lines indicating the optimal lambda. (**B**) Fivefold cross-validation for selecting adjustment parameters in the LASSO model, with each curve representing a gene. (**C**) ROC curve demonstrating the LASSO model’s diagnostic performance. (**D**) Ranking of ARDEGs based on relative importance in the random forest model. (**E**) Error rate plotted against the number of random forest trees. (**F**) ROC curve of the random forest model. (**G**) Venn diagram combining results from LASSO and random forest algorithms to identify overlapping genes. (**H**) Expression analysis of PARP1 and SDCBP in the training dataset. (**I**) Expression analysis of PARP1 and SDCBP in the validation dataset GSE147878, ***P < 0.001.

To improve the diagnostic accuracy of hub ARDEGs for asthma, we employed two machine learning models: LASSO (Fig. 5A, B) and RF (Fig. 5D, E). Both models identified PARP1 and SDCBP as hub ARDEGs (Fig. 5G, Table S14). The diagnostic efficiency was confirmed by ROC curves, with the LASSO model achieving an AUC of 0.984 (Fig. 5C) and the RF model achieving an AUC of 0.87 (Fig. 5F), indicating strong predictive capability.

Expression analysis of hub ARDEGs in both the training dataset (Fig. 5H) and the external validation dataset GSE147878 (Fig. 5I) showed significant upregulation in asthma samples.

### Verification of hub gene expression in lung tissues of asthma mice

**Fig. 6:**
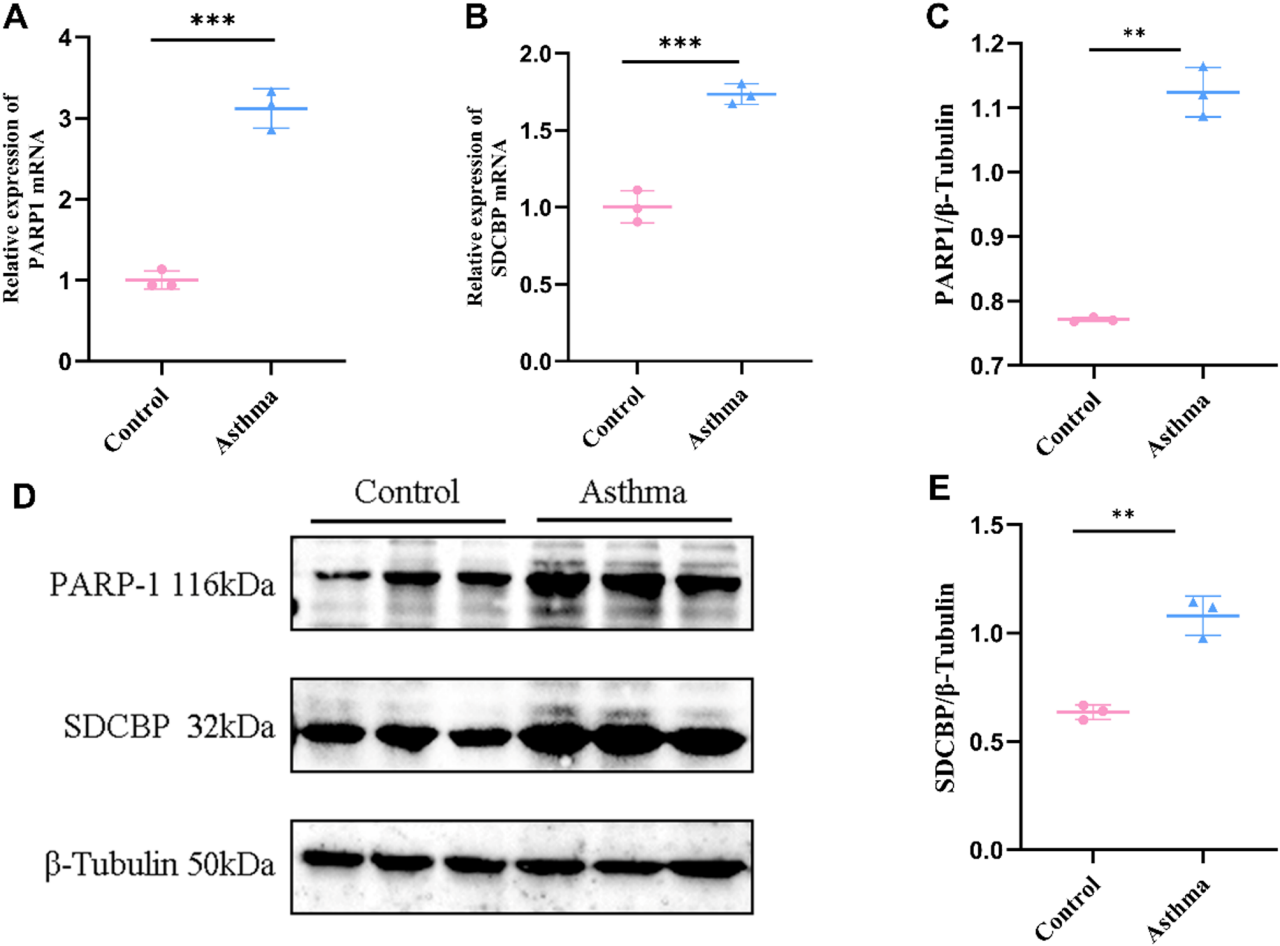
Validation of hub ARDEGs expressions in asthma mice. (**A,B**) RT-PCR analysis showing mRNA expression of PARP1 and SDCBP. (**C–E**) Representative western blot and statistical analysis of protein expression levels for PARP1 and SDCBP. Data represent three independent experiments with three mice per group *P < 0.05, **P < 0.01, ***P < 0.001.

To further verify the expression levels of PARP1 and SDCBP, we performed RT-PCR and Western blot analysis on lung tissues from OVA-induced asthma mice, compared to control mice. Consistent with our bioinformatics results, the expression of both genes was significantly upregulated in the asthma group (Fig. 6A–E).

### Construction of hub ARDEGs risk prediction model

**Fig. 7:**
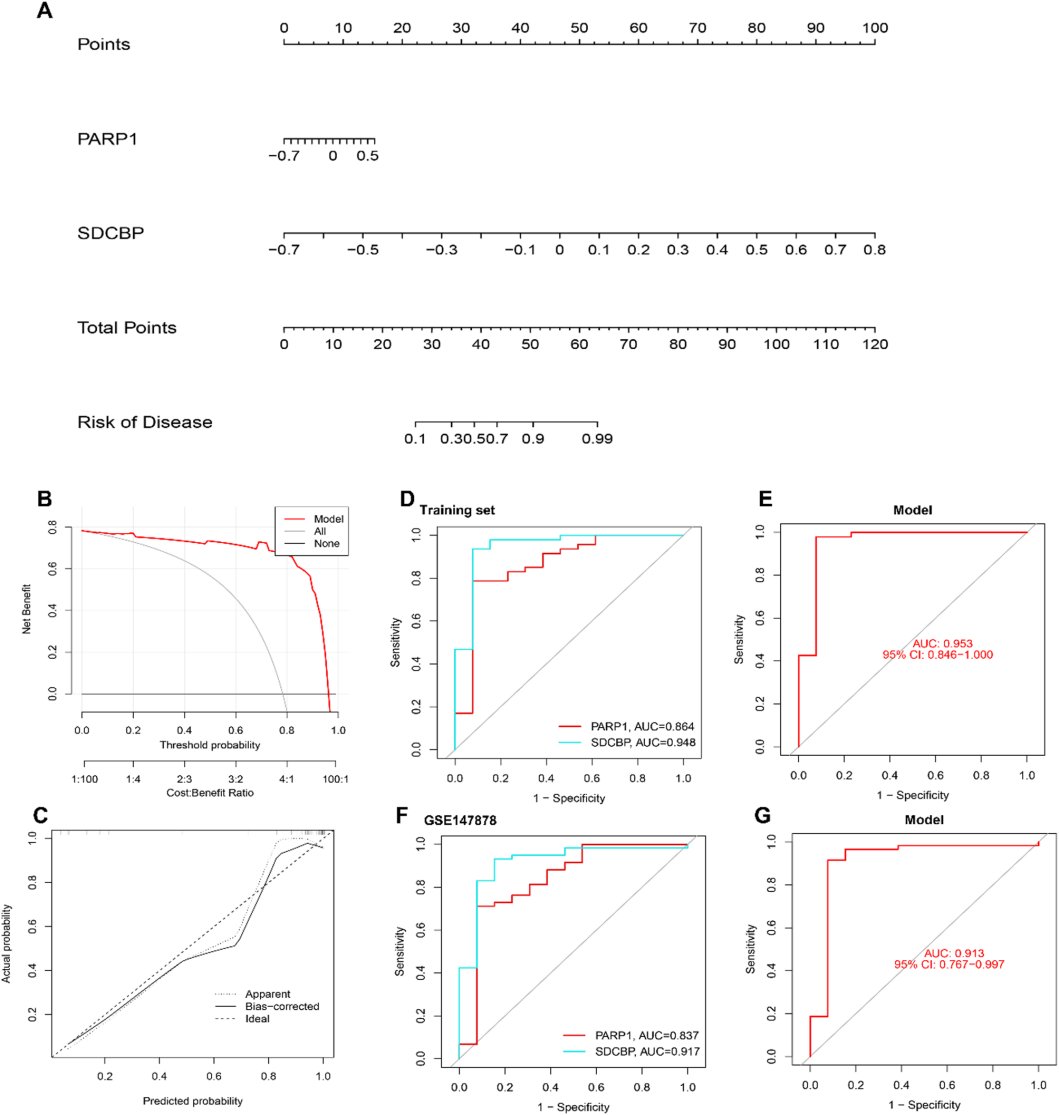
Hub ARDEGs risk prediction model. (**A**) Nomogram for asthma diagnosis based on PARP1 and SDCBP, where each gene corresponds to a score contributing to the overall risk prediction. In this nomogram, the ‘Points’ serve as a quantitative measure representing the scores assigned to each candidate gene, whereas the ‘Total Points’ reflect the overall score accumulated from all the listed genes. The predictive accuracy and clinical applicability of the nomogram were assessed in the training set. (**B**) Calibration curve assessing nomogram accuracy. (**C**) Decision curve analysis evaluating the net benefit of diagnostic decisions based on the nomogram. (**D**) ROC curves for the training set showing the diagnostic performance of PARP1 and SDCBP. (**E**) ROC curve for the training set showing the diagnostic performance of the nomogram. (**F**) ROC curves for the validation dataset GSE147878 showing the diagnostic performance of PARP1 and SDCBP. (**G**) ROC curves for the validation dataset GSE147878 showing the diagnostic performance of the nomogram.

Based on the expression patterns of the two hub ARDEGs, we developed a diagnostic nomogram (Fig. 7A). Decision curve analysis (DCA) indicated that using this model for decision-making benefited asthma patients (Fig. 7B). Additionally, the nomogram’s predictive accuracy was validated with calibration curves for the training dataset (Fig. 7C).

We performed a ROC curve analysis to evaluate their diagnostic utility. The results showed that PARP1 and SDCBP had high diagnostic accuracy for asthma in the training set, with AUC values of 0.864 and 0.948, respectively (Fig. 7D). Furthermore, the external validation dataset GSE147878 confirmed these findings, with PARP1 and SDCBP achieving AUC values of 0.837 and 0.917 (Fig. 7F). The nomogram demonstrated remarkable AUC values of 0.953 for the training set (Fig. 7E) and 0.913 (Fig. 7G) for the validation set after undergoing 5-fold cross-validation.

However, in external datasets from other tissue types other than endobronchial biopsies, the results were less robust. In the GSE64913 dataset from epithelial brushings, PARP1 and SDCBP had AUC values of 0.523 and 0.563, with a nomogram AUC of 0.567 (Supplementary Fig. 1A-C). For GSE137268 from sputum samples, the AUC values were 0.595 and 0.627, with a nomogram AUC of 0.636 (Supplementary Fig. 1D-F). In GSE27876 from peripheral blood, PARP1 and SDCBP had AUC values of 0.660 and 0.800, with a nomogram AUC of 0.860 (Supplementary Fig. 1G-I).

### The role of PARP1 and SDCBP in asthma immune microenvironment

**Fig. 8:**
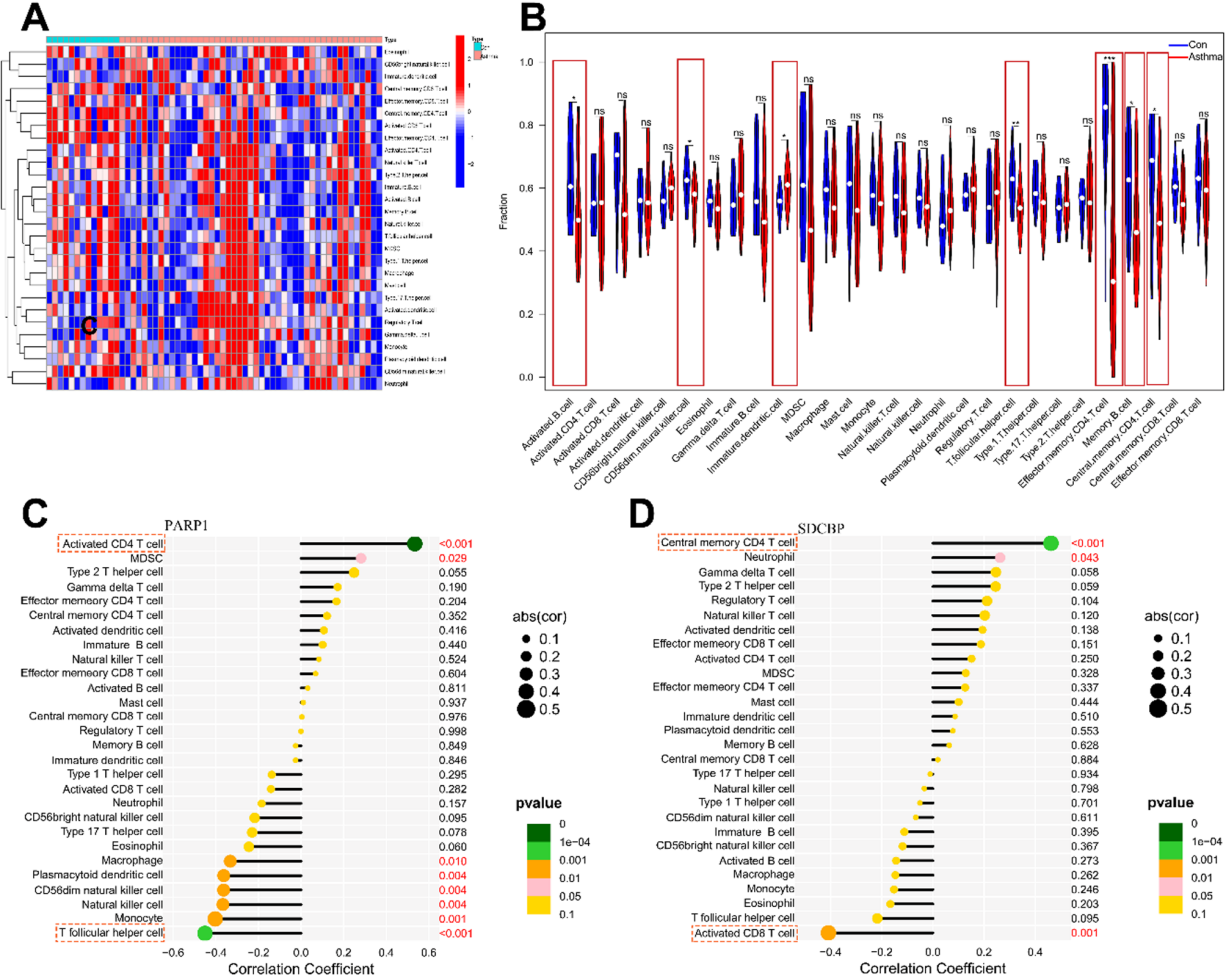
ssGSEA immune infiltration. (**A**) Heatmap depicting infiltration levels of 28 immune cells across samples. (**B**) Violin plots comparing immune cell infiltration between asthma and control samples. (**C**) Correlation Analysis between PARP1 and 28 Types of Immune Cells. (**D**) Correlation Analysis between SDCBP and 28 Types of Immune Cells. *P < 0.05, **P < 0.01, ***P < 0.001.

To explore the relationship between asthma and immune microenvironment, we conducted an in-depth analysis using the ssGSEA algorithm. The abundance of 28 immune cell types varied significantly between the asthma and control samples (Fig. 8A and B, Table S15). The relationships between distinct immune cell subsets and hub ARDEGs were revealed by correlation analysis (Fig. 8C and D). We found that PARP1 had the strongest positive correlation with activated CD4 T cells but the strongest negative correlation with T follicular helper cells. SDCBP exhibited the strongest positive correlation with central memory CD4 T cells but the strongest negative correlation with activated CD8 T cells (Fig. 8C and D).

### Enrichment analysis of hub ARDEGs

**Fig. 9:**
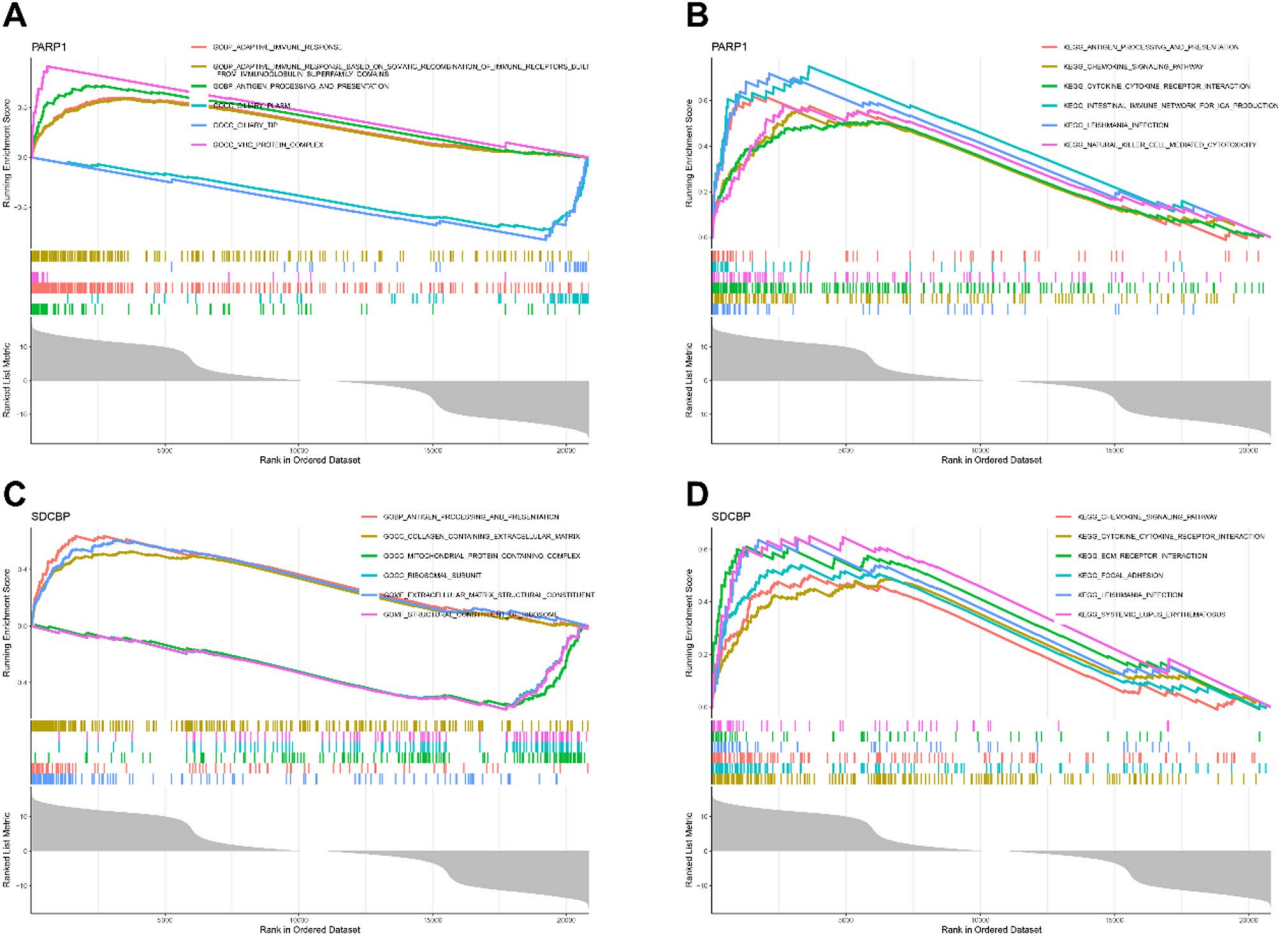
Single-gene GSEA enrichment analysis of Hub ARDEGs. GO and KEGG enrichment analyses for PARP1 and SDCBP using GSEA, displaying the top six enriched terms.

To explore the potential roles of hub ARDEGs in asthma, we performed single-gene GSEA (Table S16-S19). For PARP1, GO analysis showed upregulation in processes like “Antigen processing and presentation” and “Adaptive immune response,” with negative regulation in “Ciliary plasma” and “Ciliary tip” (Fig. 9A). KEGG pathway analysis revealed upregulation in pathways like “Antigen processing and presentation,” “Chemokine signaling pathway,” and “Natural killer cell-mediated cytotoxicity” (Fig. 9B). For SDCBP, GO analysis highlighted upregulation in processes like “Extracellular matrix structural constituent,” “Antigen processing and presentation,” and “Mitochondrial protein-containing complex,” with negative regulation in “Structural constituent of ribosome” and “Ribosomal subunit” (Fig. 9C). KEGG pathways showed upregulation in “Cytokine-cytokine receptor interaction,” “Focal adhesion,” and “Chemokine signaling pathway” (Fig. 9D).

### The miRNA-TF-mRNA regulatory network construction of hub ARDEGs

**Fig. 10:**
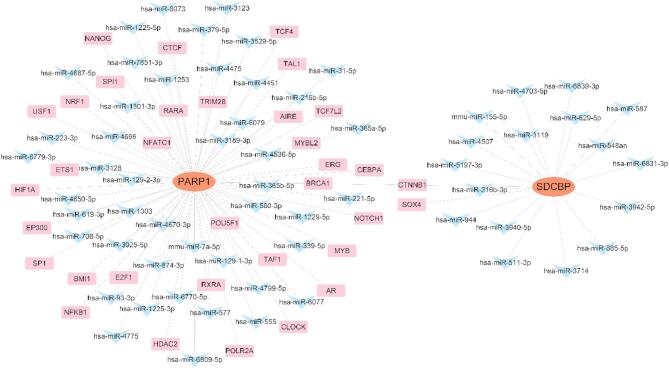
The miRNA-TF-mRNA regulatory network. Visualization of target gene mRNAs (Red Circles), miRNAs (Blue V-Shapes) and TFs (Pink Squares).

To explore regulatory mechanisms, we performed a predictive analysis on miRNAs and TFs targeting hub ARDEGs. Using Cytoscape (version 3.9.0), we created a visual representation of the complex regulatory network, which included 62 miRNAs, 35 TFs, and 2 genes, resulting in 97 regulatory interactions between miRNAs, TFs, and mRNAs (Fig. 10, Table S20).

## Discussion

Asthma presents a significant clinical challenge due to its high prevalence and chronic nature^4^. Investigating its underlying mechanisms and developing targeted therapies have long been research priorities. One potential mechanism involved in asthma pathogenesis is anoikis, a specialized form of apoptosis that occurs when cells detach from the ECM. This process triggers either mitochondrial-dependent intrinsic pathways or death receptor-mediated extrinsic pathways^19^. However, the exact role of anoikis in asthma and its contribution to airway remodeling remain unclear. This study used bioinformatics analysis to investigate anoikis-related biomarkers in asthma endobronchial biopsies and explore the underlying mechanisms involved.

Firstly, GO and KEGG enrichment analyses revealed that DEGs were primarily involved in transcriptional regulation in response to hypoxia, stress, oxidoreductase activity, and key pathways such as the cGMP-PKG signaling pathway and TGF-beta signaling pathway, aligning with previous research^20,21^. GSEA indicated that the hub ARDEGs PARP1 and SDCBP were involved in antigen processing and presentation, adaptive immune responses, and extracellular matrix structural organization, with enriched pathways related to the chemokine signaling pathway and cytokine-cytokine receptor interactions. These findings suggest that PARP1 and SDCBP play crucial roles in the immune and inflammatory responses associated with asthma, highlighting their potential as diagnostic biomarkers and therapeutic targets.

Poly ADP-ribose polymerase-1 (PARP1) is a key member of the PARP protein family, involved in DNA repair and cellular stress responses^22^. It plays a significant role in the pathogenesis of asthma. Studies have shown that the expression and activity of PARP1 are increased in lung tissues from asthma mouse models and patients^23,24^. In asthma, excessive reactive oxygen and nitrogen species (ROS/RNS) production by inflammatory cells causes DNA damage and activates PARP1. PARP1 is crucial for maintaining signal transducer and activator of transcription 6 (STAT6) integrity, which regulates GATA binding protein 3 (GATA3) and IL-5 expression driving the immune response, especially through splenic CD4 T cell maturation^25,26^. PARP1 also regulates vimentin expression, influencing the NLR family pyrin domain containing 3 (NLRP3) inflammasome and promoting airway remodeling^27^. overexpression of PARP1 has been shown to promote apoptosis and inhibit the proliferation of excessive human airway smooth muscle cells, a key feature of airway remodeling in asthma^28^. However, the exact mechanisms through which PARP1 influences anoikis and its role in asthma are yet to be fully understood. This finding implies its potential application as an anoikis related biomarker for asthma. Syndecan binding protein (SDCBP), also called melanoma differentiation associated protein 9 (MDA − 9) or Syntenin − 1, was identified as a vital mediator connecting syndecan signaling to the cytoskeleton^29^. It is a PDZ - domain protein with many interaction partners, and plays a key role in transmembrane receptor trafficking, cell adhesion, neuronal synapse function, and tumor metastasis^30^. Emerging evidence indicates that SDCBP participates in apoptosis regulation. In cancer, SDCBP reduces apoptosis and promots tumor progression and survival^30–32^. Conversely, in a cardiac hypoxia - reoxygenation (H/R) model, stress upregulates SDCBP expression, which promotes cardiomyocyte apoptosis^33^. In the context of anoikis, previous research showed that SDCBP aids glioma stem cell survival by facilitating protective autophagy, phosphorylating B-cell lymphoma 2 (BCL2), and regulating the epidermal growth factor receptor (EGFR) signaling pathway, thus inhibiting anoikis^34^. However, the role of SDCBP in asthma is mostly unknown, and no studies have explored its impact on anoikis in asthma. Intriguingly, the increased SDCBP expression in endobronchial biopsies tissues of asthma patients implies its potential involvement in cell survival and apoptotic resistance mechanisms.

Secondly, our findings showed that hub ARDEGs have strong diagnostic potential for asthma and were significantly upregulated in endobronchial biopsies samples from both the training set and validation set GSE147878. However, external validation on datasets from other tissue sources revealed that AUC values for PARP1 and SDCBP were ≤ 0.80, indicating suboptimal diagnostic performance. These discrepancies may arise from tissue-specific factors, including cellular composition and technical limitations. (1) Airway epithelial brushings primarily consist of epithelial cells^35^, while endobronchial biopsies include a broader mix of epithelial, immune, and stromal cells^36^, likely leading to higher marker expression. Specifically, PARP1, which is involved in recruiting and activating CD4^+^T cells, eosinophils, and dendritic cells during Th2 inflammation in asthma^25^, is likely more expressed in biopsies due to the involvement of immune cells. SDCBP has limited evidence supporting its role in asthma and requires further study. (2) Sputum mainly composed of mucus and respiratory secretions and contains fewer immune and stromal cells^37^, making it difficult to detect non-secreted proteins like PARP1 and SDCBP. Additionally, sputum samples are prone to contamination by oral secretions, introducing salivary enzymes that can lead to RNA degradation, thus impairing detection^38^. (3) Peripheral blood samples did not show the same upregulation of PARP1 and SDCBP as endobronchial biopsies, suggesting that systemic immunity does not reflect local airway inflammation. However, the diagnostic model based on peripheral blood showed a higher AUC of 0.86, indicating better overall performance. While this model may be valuable for non-invasive diagnostics, caution is needed when applying it to certain tissue sources. These findings stress the importance of sample source in diagnostic accuracy, and future models should be validated for each specific tissue type.

Thirdly, we established an asthma mouse model using OVA induction. Western blot and RT-PCR were used to further validate the expression of PARP1 and SDCBP in asthma. These results aligned with our bioinformatics analysis and further reinforced the robustness of our findings.

Fourthly, we examined the correlations between hub ARDEGs and immune cell subsets in the asthma microenvironment, revealing potential roles in immune response. Our analysis revealed that PARP1 was positively correlated with activated CD4 T cells, consistent with previous studies, suggesting its role in regulating STAT6, GATA3, and IL-5 in immune responses^25,26^. In contrast, PARP1 was negatively correlated with T follicular helper cells, whose function is regulated by transcription factors like BCL6^39^. PARP1 may inhibit T follicular helper cell generation by modifying BCL6, thereby reducing IgE production by B cells in germinal centers. This suggests that PARP1 helps balance CD4 T cell and T follicular helper cell functions, potentially influencing asthma modulation. SDCBP showed a positive correlation with central memory CD4 T cells, likely promoting their activation and differentiation to sustain immune responses in asthma^40^. Conversely, SDCBP negatively correlated with activated CD8 T cells, which can damage the airway epithelium and worsen inflammation. By reducing CD8 T cell activation, SDCBP inhibits the conversion of these cells into Tc2 cells, which produce Th2-type cytokines^41^. Therefore, SDCBP plays a dual role in asthma by promoting memory CD4 T cell responses while limiting activated CD8 T cell activity.

Lastly, Single-gene GSEA and the miRNA-TF-mRNA regulatory network analysis revealed the potential roles of hub ARDEGs in asthma. In GSEA, PARP1 and SDCBP were associated with immune-related processes like ‘Antigen processing and presentation’ and ‘Adaptive immune response,’ emphasizing their role in immune regulation in asthma. These findings offer new insights into asthma’s molecular mechanisms. Additionally, the identified miRNAs and TFs form a complex regulatory network with hub ARDEGs, suggesting potential targets for further research.

However, there are several limitations. First of all, the use of public transcriptomic data limits access to clinically relevant information, such as comorbidities and treatment history, which could introduce confounding variables. Additionally, the small, potentially homogeneous sample size may not fully represent the genetic and immune diversity in asthma patients, highlighting the need for larger studies. While the database analysis was thorough, limited experimental validation calls for further studies to confirm the functional roles of the identified genes. Specifically, future experiments will explore the mechanisms underlying anoikis in asthma, and examine how the inhibition of PARP1 and SDCBP affects anoikis and airway inflammation in asthma mouse models.

## Conclusion

To sum up, we evaluated the association between ARGs and asthma using bioinformatics and machine learning, and we created a predictive model to identify sensitivity in asthma patients, particularly in endobronchial biopsies samples. This study provides new insights into the biological mechanisms linking anoikis and asthma, deepening our understanding of its role in asthma pathogenesis and opening up new avenues for treatment strategies and personalized medicine.

## Electronic supplementary material

Below is the link to the electronic supplementary material.

Supplementary Material 1

Supplementary Material 2

Supplementary Material 3

## Data Availability

The data supporting the findings of this study are publicly available in the Gene Expression Omnibus at https://www.ncbi.nlm.nih.gov/geo/, with reference numbers GSE143303, GSE147878, GSE64913, GSE137268, and GSE27876; the Harmonizome at https://maayanlab.cloud/Harmonizome/; and the GeneCards database at https://www.genecards.org/. Further inquiries can be directed to the corresponding authors.

## Abbreviations

GINA: Global initiative for asthma
ECM: Extracellular matrix
MMP9: Matrix metalloproteinase 9
GEO: Gene expression omnibus
ARGs: Anoikis-related genes
DEGs: differentially expressed genes
GO: Gene Ontology
KEGG: Kyoto encyclopedia of genes and genomes
GSEA: Gene set enrichment analysis
WGCNA: Weighted gene co-expression network analysis
MAD: Median absolute deviation
TOM: Topological overlap matrix
Mes: Module eigengenes
MM: Module membership
GS: Gene significance
ARDEGs: Anoikis-related differentially expressed genes
LASSO: Least absolute shrinkage and selection operator
RF: Random forest
RFE: Recursive feature elimination
ROC: Receiver operating characteristic
OVA: Chicken egg chicken
SDS-PAGE: Odium dodecyl sulfate-polyacrylamide
PVDF: Polyvinylidene fluoride
ssGSEA: Single sample gene set enrichment analysis
GSEA: Gene set enrichment analysis
TFs: Transcription factors
BP: Biological processes
CC: Cellular components
MF: Molecular functions
PARP1: Poly ADP-ribose polymerase-1
ROS/RNS: Reactive oxygen and nitrogen species
STAT6: Signal transducer and activator of transcription 6
GATA3: GATA binding protein 3
NLRP3: NLR family pyrin domain containing 3
SDCBP: Syndecan binding protein
MDA-9: Melanoma differentiation associated protein 9
H/R: Hypoxia-reoxygenation
BCl2: B-cell lymphoma 2
EGFR: Epidermal growth factor receptor
