# Supplementary material for: Anoikis-related biomarkers PARP1 and SDCBP as diagnostic and therapeutic targets for asthma
Source: Sci Rep. 2025 Jul 9;15:24779. doi: 10.1038/s41598-025-09979-9 (PMC12241600; doi:10.1038/s41598-025-09979-9)

REPEAT1

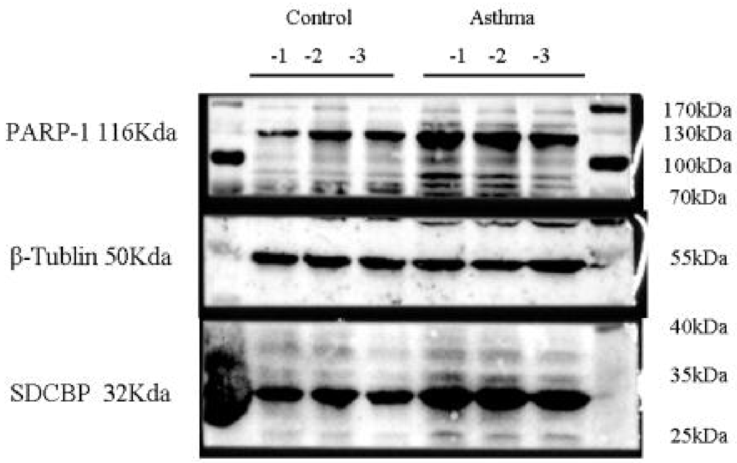

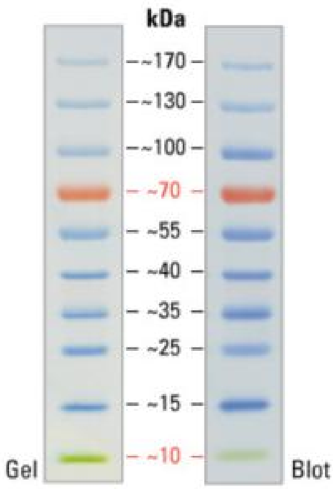

Whole membrane 1
(uncutted)

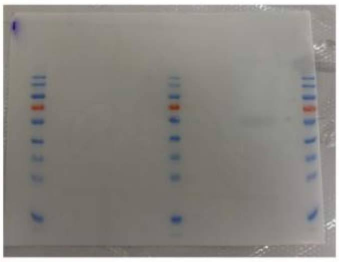

Whole membrane 3
(marker)

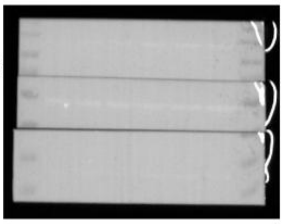

Whole membrane 2
(cutted,
choose the right side)

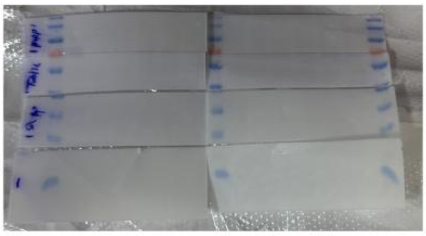

Whole membrane 4

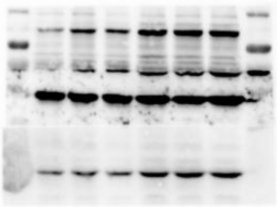

REPEAT2 and 3 of PARP1

---- Because SDCBP has a lot of spurious bands and the main bands don't show up clearly, we don't look at the results of SDCBP, but only at PARP1

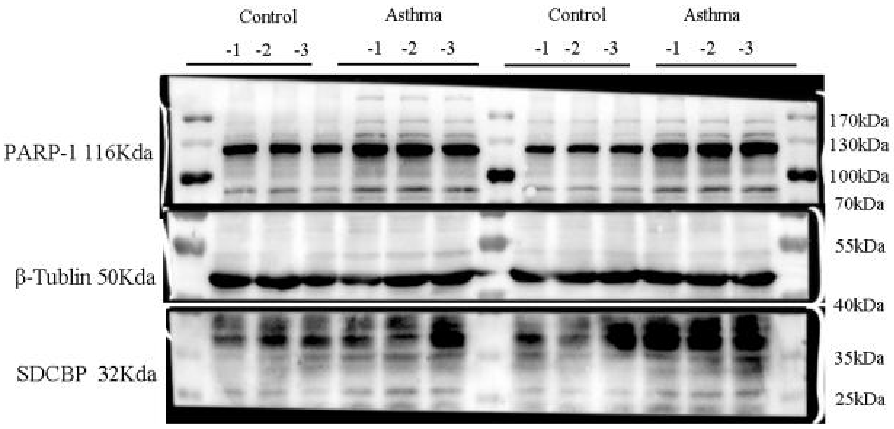

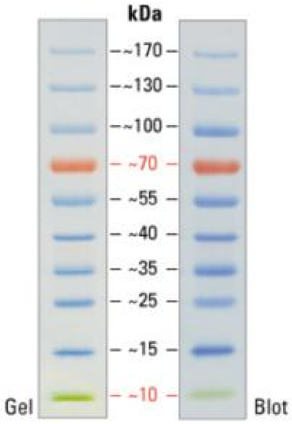

Whole membrane 1
(uncutted)

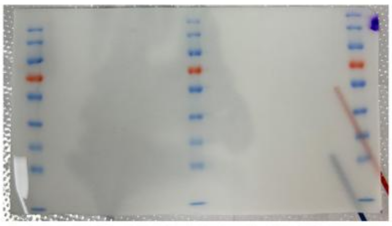

Whole membrane 3
(marker)

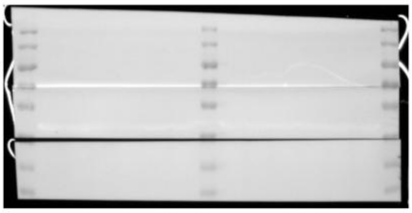

Whole membrane 2
(cutted)

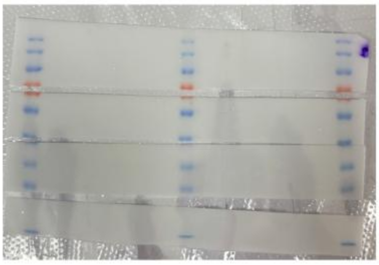

Whole membrane 4

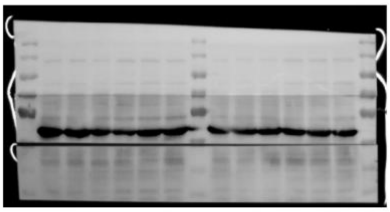

REPEAT2 and 3 of SDCBP

---- Because PARP1 has a lot of heterozygous bands and the main bands don't show up clearly, we don't look at the PARP1 results, only at SDCBP

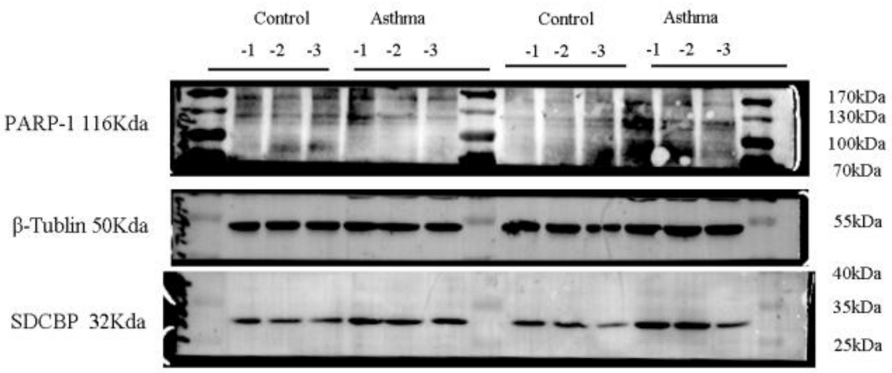

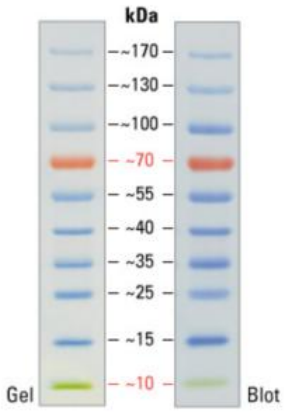

Whole membrane 1 (uncutted)

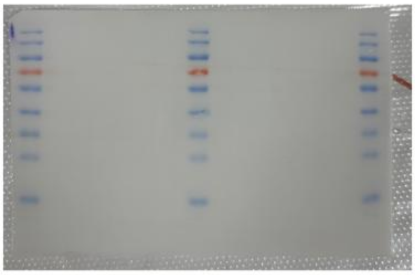

Whole membrane 3 (marker)

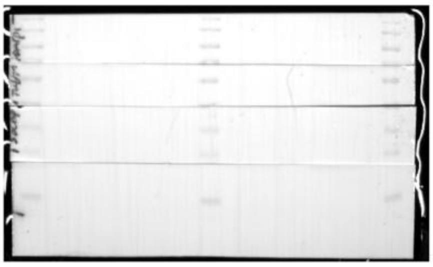

Whole membrane 2 (cutted)

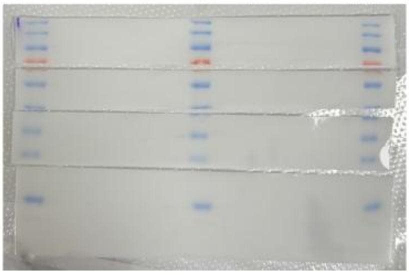

Whole membrane 4

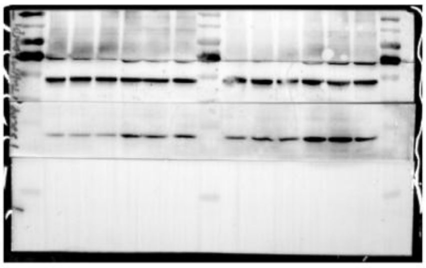

Supplement: Supplementary file 1 — Supplementary Material 1 [file 41598_2025_9979_MOESM1_ESM.pdf]
