# Supplementary material for: Anoikis-related biomarkers PARP1 and SDCBP as diagnostic and therapeutic targets for asthma
Source: Sci Rep. 2025 Jul 9;15:24779. doi: 10.1038/s41598-025-09979-9 (PMC12241600; doi:10.1038/s41598-025-09979-9)
Supplement: Supplementary file 2 — Supplementary Material 2 [file 41598_2025_9979_MOESM2_ESM.pdf]

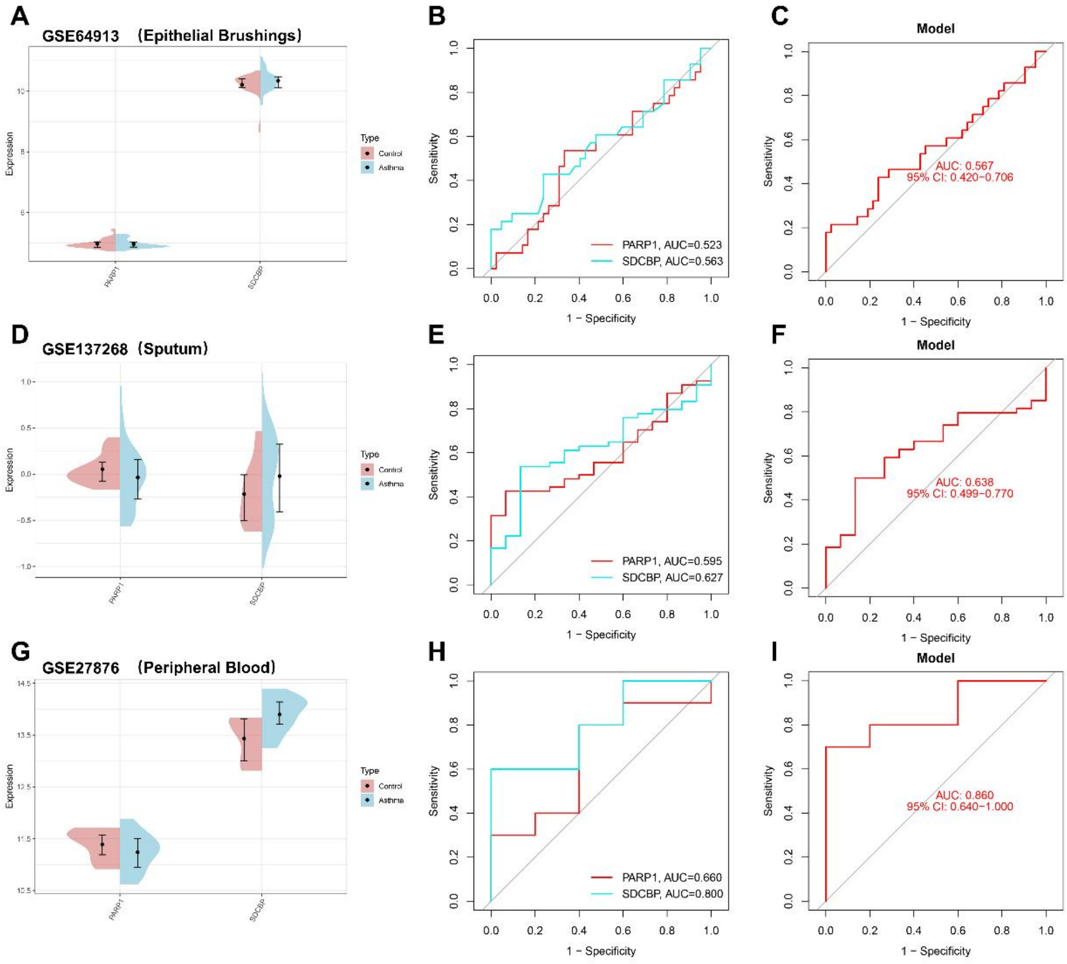

Evaluation of hub ARDEGs expression and nomogram in external datasets. (A) Expression of PARP1 and SDCBP in asthma and control groups in GSE64913. (B) ROC curves for GSE64913 showing the diagnostic performance of PARP1 and SDCBP. (C) ROC curves for GSE64913 showing the diagnostic performance of the nomogram. (D) Expression of PARP1 and SDCBP in asthma and control groups in GSE137268. (E) ROC curves for GSE137268 showing the diagnostic performance of PARP1 and SDCBP. (F) ROC curves for GSE137268 showing the diagnostic performance of the nomogram. (G) Expression of PARP1 and SDCBP in asthma and control groups in GSE27876. (H) ROC curves for GSE27876 showing the diagnostic performance of PARP1 and SDCBP. (I) ROC curves for GSE27876 showing the diagnostic performance of the nomogram. $^{**}P < 0.01$. $^{***}P < 0.001$.
